# Spontaneous In-Source Fragmentation Reaction Mechanism and Highly Sensitive Analysis of Dicofol by Electrospray Ionization Mass Spectrometry

**DOI:** 10.3390/molecules28093765

**Published:** 2023-04-27

**Authors:** Jun Xie, Yage Guo, Yongqiang Ma, Hongyun Jiang, Lan Zhang, Liangang Mao, Lizhen Zhu, Yongquan Zheng, Xingang Liu

**Affiliations:** 1 State Key Laboratory for Biology of Plant Diseases and Insect Pests, Institute of Plant Protection, Chinese Academy of Agricultural Sciences, Beijing 100193, China; xxjj2410@foxmail.com (J.X.);; 2 Department of Applied Chemistry, College of Science, China Agricultural University, Beijing 100193, China; 3 Shandong Engineering Research Center for Environment-Friendly Agricultural Pest Management, College of Plant Health and Medicine, Qingdao Agricultural University, Qingdao 266109, China

**Keywords:** dicofol, in-source fragmentation, organic pollutant, HPLC-MS/MS, ESI

## Abstract

Although dicofol has been widely banned all over the world as a kind of organochlorine contaminant, it still exists in the environment. This study developed a high-performance liquid chromatography coupled with mass spectrometry (HPLC-MS/MS) detection technique for dicofol, an environmental pollutant, for the first time using in-source fragmentation. The results confirmed that *m*/*z* 251 was the only precursor ion of dicofol after in-source fragmentation, and *m*/*z* 139 and *m*/*z* 111 were reasonable product ions. The main factors triggering the in-source fragmentation were the H^+^ content and solution conductivity when dicofol entered the mass spectrometer. Density functional theory can be used to analyze and interpret the mechanism of dicofol fragmentation reaction in ESI source. Dicofol reduced the molecular energy from 8.8 ± 0.05 kcal/mol to 1.0 ± 0.05 kcal/mol, indicating that the internal energy release from high to low was the key driving force of in-source fragmentation. A method based on HPLC-MS/MS was developed to analyze dicofol residues in environmental water. The LOQ was 0.1 μg/L, which was better than the previous GC or GC-MS methods. This study not only proposed an HPLC-MS/MS analysis method for dicofol for the first time but also explained the in-source fragmentation mechanism of compounds in ESI source, which has positive significance for the study of compounds with unconventional mass spectrometry behavior in the field of organic pollutant analysis and metabonomics.

## 1. Introduction

The analysis of organic compounds in a high-performance liquid chromatography-tandem triple quadrupole mass spectrometry (HPLC-MS/MS) equipped with an electrospray ionization (ESI) source is intended to quantify the mass-charge ratio (*m*/*z*) strength of the analyte in order to determine its concentration in the matrix. The analyte is separated from a mixture by an HPLC and then ionized by ESI. The interest ion is fragmented by either low-energy collision-induced dissociation (CID) with the inert gas or the reaction of the reagent radical, providing an MS/MS spectrum to analyze [1]. This technique is widely employed as a mature method for monitoring trace environmental pollutants such as pesticides, veterinary medicines, drugs, and other persistent organic pollutants (POPs) [2,3]. In-source fragmentation (ISF) is a natural phenomenon in pre-ionization analysis techniques such as ESI source or atmospheric pressure chemical ionization (APCI) source [4,5,6]. Before CID, the analytes dissociate during the ionization phase. In contrast to adducts, the precursor ions of in-source fragmentation are composed of missing large molecule fragments. Notably, *m*/*z* values are typically specific to each substance. Positively, in-source fragmentation has contributed to the structural identification of complex compounds such as proteins, peptides, and polysaccharides [7,8]. However, in-source fragmentation has long been regarded as a significant negative factor in the quantitative analysis of compounds, resulting in a poor ability to detect target compounds using ESI-MS. The resulting ineffective ionization, low abundance, and excessive fragmentation of analytes reduce sensitivity and reproducibility. In the quantitative analysis of compounds, however, in-source fragmentation is rarely taken seriously. Still, more than 80% of molecules dissociate readily into multiple fragments at low energies [9,10].

Both in-source fragmentation and CID fragmentation are caused by the cleavage of chemical bonds. Recent studies have confirmed the application value of in-source fragmentation for quantitative analysis of compounds based on *m*/*z* values. For example, He et al. [9,11] suggested that the abundance and availability of in-source fragment ions of some pesticides were higher than that of common precursor ions such as [M + H]^+^ or [M + NH_4_]^+^, which means that more high-abundance characteristic ions may be selected as the precursor ions of the analyte. This also suggests that compounds previously considered difficult to detect in pre-ionization analysis instruments are likely to have high ion abundance in in-source fragmentation, which provides the possibility of high-sensitivity analysis. Many studies have used the specificity of ISF ions to detect azadirachtin, 3-tigloylazadirachtol, abamectin, and other compounds [12,13].

However, environmental and food studies have focused only on whether compounds can be effectively analyzed, and relatively few of them focus on in-source fragmentation more deeply. Studies have found that different types of compounds produce different forms of ISF ions. In general, a molecule dissociates by group removal and becomes a natural ionic compound directly ([M] → [M − N]^+^/[M − N]^−^), such as insecticides dimethoate and azinphos-entyl [9]. Other molecules formed ISF ions by in situ or ex situ protonation or ammonium ionization after the group leaves ([M] → [M − N + H]^+^/[M − N + NH_4_]^+^). Current methods of quantifying analytes using ISF ions still face the disadvantage of insufficient stability. According to the principle of ionization in a mass spectrometer, this is the trouble of chemical reaction and reaction stability of organic molecules in ESI source. To understand these differences, we focused on the mechanism of the reaction of a compound dissociation in the ESI source.

Dicofol (2,2,2-trichloro-1,1-bis (4-chlorophenyl) ethanol, CAS 115-32-2), as one of the major classes of organochlorine pesticides, was used on a worldwide variety of fruit, ornamentals, and field crops previously [14,15]. Despite the agricultural benefits of dicofol, numerous studies have demonstrated its toxicity, capacity for endocrine disruption, and carcinogenicity via the biological accumulation effect [16,17]. Stockholm Convention identified dicofol as a persistent organic pollutant (POP) that many countries banned or limited its use [14]. Due to its exceptional chemical stability, dicofol has the inherent property of a long residue. It is detected in air, water, sediment, soil, and even human breast milk throughout the world, including Antarctica [18,19,20,21,22]. Ongoing global monitoring of dicofol in the environment is still necessary to control risks better. With its similarity of the molecular structure to dichlorodiphenyltrichloroethane (DDT), dicofol has previously been analyzed only by gas chromatography coupled to electron capture detector (GC-ECD) or gas chromatography-mass spectrometry (GC-MS) [23,24], which with disadvantages of low sensitivity, high purification requirement, troublesome extraction process, and long analysis time.

This research investigated the mass spectrometry behavior of dicofol. We focused on the occurrence frequency and chemical mechanism of the fragmented dicofol in the ESI source. The results provide direct and conclusive evidence that effective control of the in-source fragmentation reaction in the ESI source not only increased analytical sensitivity but also resolved a technical issue with the HPLC-MS/MS analysis of dicofol. This research may encourage the expansion of analytical chemistry, omics, and mass-spectrometry research fields.

## 2. Results and Discussion

### 2.1. Confirmation of In-Source Fragmentation in ESI Source

**Figure 1:**
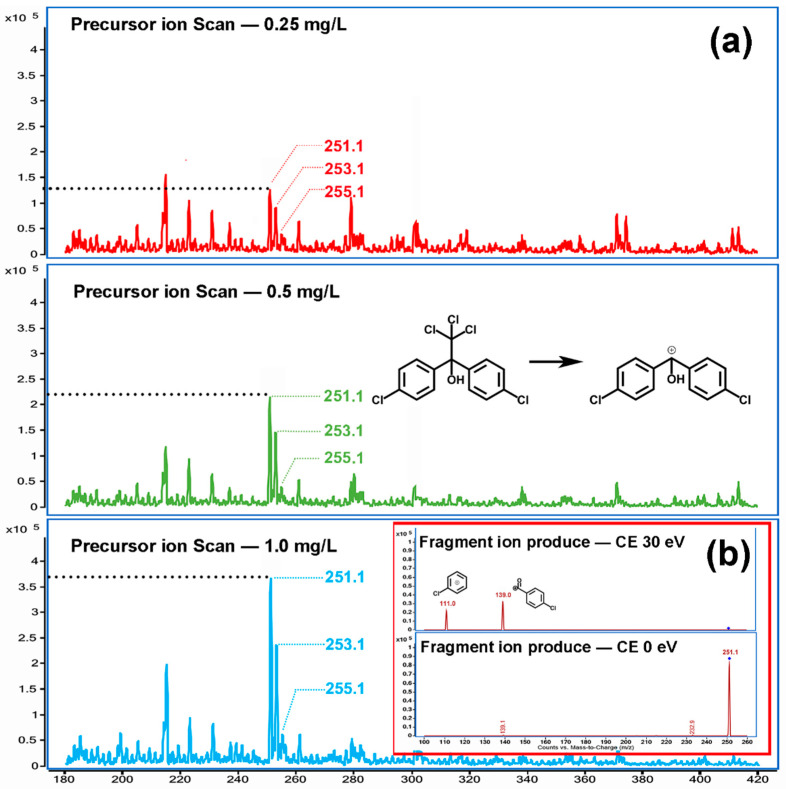
Dicofol signal in mass spectrometer. (**a**) the abundance of ions *m*/*z* 251, 253, and 255 was positively correlated with the sample concentration, and the difference 117 was the same as the mass-to-charge ratio of trichloromethyl. It was inferred that this signal might be the in-source fragmentation ion produced by dicofol losing trichloromethyl. (**b**) The product ions of CID included only *m*/*z* 111 and 139, which were both fragment ions of *m*/*z* 251. The existence of in-source fragmentation reaction was confirmed by these mass spectrometric behaviors.

In order to reduce the interference of impurities, a solution of three concentration levels of 500, 100, and 50 μg/L was prepared with LC-MS grade methanol as solvent. The solutions were scanned by a mass spectrometer in liquid-mass mode, allowing the mixture in the standard solution to be fully separated. As shown in Figure 1, the formula for dicofol is C_14_H_9_Cl_5_O, and the optimal single isotope exact mass is 368. All desired optimal single isotope ions [M + H]^+^ (*m*/*z* 369), [M + NH_4_]^+^ (*m*/*z* 386), [M + Na]^+^ (*m*/*z* 391) and did not appear in the mass spectrogram. However, the response of the mass spectrogram signal *m*/*z* 251 was positively correlated with the standard solutions’ concentration, which indicated that the molecular ion was highly correlated with dicofol (Figure S2). *m*/*z* 251, *m*/*z* 253, and *m*/*z* 255 are three continuous mass spectral signals with a mass charge ratio difference of two.

The abundance ratio of 9:6:1 suggests that three signals come from the same ion with two chlorine atoms (Figure S3). The molecular mass difference between *m*/*z* 251 and the dicofol molecule is 117, equal to the formula mass of trichloromethyl, a chemical group of dicofol molecule, which indicated that *m*/*z* 251 might be a positive ion produced by dicofol striped the trichloromethyl and the H atom on the benzyl carbon. The *m*/*z* 251, as the precursor ion, was then fed into the collision cell of a triple quadrupole mass spectrometer (QQQ) and collision-induced dissociation (CID) with helium. Two reasonable fragment ions were observed. The first, *m*/*z* 111, was presumed to be a positive ion of the chlorobenzene absent the *para* site H atom. The second, *m*/*z* 139, was presumed to be a positive ion of the 4-chlorobenzaldehyde absent the H atom in the aldehyde group (Figure S4).

According to the above mass spectrum characteristics, dicofol in the ESI source of the mass spectrometer underwent in-source fragmentation, resulting in a fragmentation ion devoid of trichloromethyl. The fragmentation ion then collides with and dissociates into the precursor ion. The optimal ionization parameter is finally confirmed: the Fragmentor was 112 V; the Collision Energy was 41 V for ion 111 *m*/*z* and 19 V for ion 139 *m*/*z*; and the ion 139 *m*/*z* was used as a quantitative ion.

### 2.2. Influences of Critical Source Parameters

By further optimizing the source parameters, dicofol exhibited a high degree of in-source fragmentation, making it simpler to analyze with HPLC-MS/MS (ESI source). This effectively increased the results’ sensitivity and prevented false negatives. Drying gas is utilized to liberate desolvated ionized compounds from atomized droplets, so temperature and flow rate can influence desolvation efficiency. In general, a drying temperature that is too low will result in incomplete desolvation, while a drying temperature that is too high may result in premature pyrolysis of the target compound. In addition, a low flow rate reduces desolvation efficiency, whereas a high flow rate may cause droplets and ions to be blown away.

In this study, when the gas temperature was adjusted from 200 °C to 400 °C, the sensitivity of dicofol increased first and then decreased, reaching the peak at 325 °C (Figure S5). Gas flow can be adjusted up to 15 L/min, but dicofol reaches its best at 10 L/min (Figure S6). Nebulizer is used to spray inert gas to create nebulization pressure on the liquid flow in the ESI nozzle. Nebulizer can be optimized in the 0 to 60 psi range, and dicofol sensitivity is optimal when this parameter is set to 50 psi (Figure S7). This trend is because too small nebulization pressure is difficult to form favorable atomization conditions and effective droplets. At the same time, too large pressure can also make small droplets blow away.

Sheath gas is a columnar curtain of gas that is sprayed downward around the nozzle. The primary purpose is to aid in the desolvation process and limit the escape of charged droplets and ions. Therefore, the effect of sheath gas temperature and flow rate on mass spectral sensitivity is entirely positive. So, in this study, the maximum values, 400 °C and 12 L/min, are finally set (Figures S8 and S9). Next, the nozzle voltage is applied to the nebulizer to electrify the liquid prior to nebulization, similar to electrophoresis. In our study, the detection sensitivity of the dicofol increased with the increase of the nozzle voltage, so the maximum value of 2000 V was selected as the set value (Figure S10). Finally, capillary voltage is applied to the inlet end of the capillary, resulting in a high and stable ionization efficiency. However, neither an excessively high nor an inadequate capillary voltage is conducive to stable ionization. Therefore, 1800 V was chosen as the set value in our study (Figure S11).

Consider the mass spectrometric behavior of the dicofol study above. The source parameters should be set as follows: gas temperature 325 °C, gas flow 15 L/min, nebulizer 50 psi, sheath gas temperature 400 °C, sheath gas flow 12 L/min, nozzle voltage 2000 V, and capillary 1800 V.

### 2.3. Influence of Chromatographic Conditions

In order to obtain a signal with greater sensitivity from mass spectrometry, it is essential to establish good chromatographic separation parameters. Therefore, the HPLC elution parameters of dicofol were optimized in this study, including the type of mobile phase and the ratio of organic phase to inorganic phase. As organic phases, acetonitrile and methanol were compared in a binary solvent system. When methanol was used in the organic phase, the sensitivity of dicofol was eight times greater than when acetonitrile was used. Dicofol shows a high octanol-water partition coefficient (logKow, 4.28). Thus, it requires a high proportion of organic phase for elution [25], resulting in a low conductivity of nozzle droplets due to low water content. In previous studies, it was believed that the bulk conductivity limits the stability regime of the Taylor cone-jet. When the conductivity is below a critical value, the droplet generation rate, which is dependent on charge separation, is insufficient for a stable cone-jet. Due to the greater conductivity of water–methanol mixtures in comparison to water–acetonitrile, the cone-jet stability islands of water–methanol mobile phases are larger, and the ionization efficiency is increased [26,27].

Furthermore, 0.02% formic acid, 0.2% formic acid, pure water, 2.6 mM (0.02%) ammonium acetate, and 0.02% ammonia solution were used in the aqueous phase to observe the sensitivity of dicofol, respectively. The results showed that the mass spectrum signal of dicofol at the same concentration was 0.2% formic acid > 0.02% formic acid > ammonium acetate > ammonia solution > pure water (Figure S12). In addition, introducing electrolytes to the aqueous phase would enhance the conductivity and make the cone-jet stable. H^+^ content may play a critical role in the occurrence of in-source fragmentation, which will be discussed in Section 3.4.

An interesting phenomenon is that when dicofol is eluded, the sensitivity is always best when the organic phase percentage is 85%, regardless of methanol or acetonitrile. According to the discussion above, it may be related to the desolvation process and H^+^ content. On the other hand, when the proportion of the organic phase is lower than 85%, the high water content in the mobile phase reduces the ionization efficiency of nozzle droplets, which may be caused by (1) the coulombic fission (explosion) is restrained by the increase of surface tension of nozzle droplet; (2) the non-volatility of water reduces the desolvation efficiency and limits the ionization efficiency; and (3) the precursor ions may form water cluster ions.

According to the above research conclusions, the HPLC final parameters were set as follows: 95% aqueous phase from 0 to 0.5 min; 15% aqueous phase from 1 to 3.5 min; 95% aqueous phase from 3.6 to 4 min. The flow rate was 0.3 mL/min. The aqueous and organic phase was 0.2% formic acid and methanol, respectively. Under these conditions, the chromatographic retention time of dicofol was 2.88 min.

### 2.4. Electron Population Analysis

The proton reaction in the in-source fragmentation of dicofol was explored by Density Functional Theory [28]. The density functional theory is an effective method for studying a molecule’s reactivity and selectivity. Fukui function, which indicates the propensity for electron density to deform at a given position upon accepting or donating electrons, is one of the most fundamental and widely used techniques for predicting local reaction parameters [29,30]. Fukui function provides information about the electrophilic/nucleophilic power of any atomic site in a molecule. According to the definition of the dual descriptor, if Df(r) > 0, then the site is prone to nucleophilic attack, and the site is prone to electrophilic attack when Df(r) < 0.

**Figure 2:**
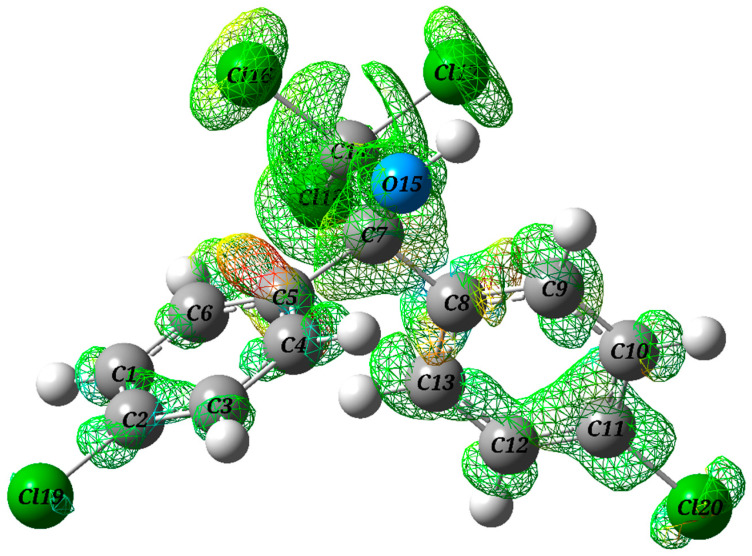
Molecular electrostatic potential map of the dicofol.

The electron population on the surface of dicofol molecules was analyzed using the Gaussian 16 program and density functional theory. Figure 2 is a molecular electrostatic potential map illustrating the propensity of each site to be electrophilically attacked. The map is a 3D plot of electrostatic potential mapped onto an electron density surface, depicting the size, shape, charge density, and chemical reactivity site of the molecules. The colorful mesh structure represents an abundance of electrons. The closer a site is to red on the color gradient from blue to green to yellow to red, the more susceptible it is to electrophilic attack. The C5 carbon of the dicofol molecule has the highest electron density. In the 3D plot, the mesh is depicted in red, representing the most susceptible site to proton electrophilic attack, namely the initial step of the in-source fragmentation reaction.

### 2.5. Mechanisms of Bond Cleavage and In-Source Fragmentation

The precursor ion *m*/*z* 251.1 is a naturally positive ion after a bond rupture that has not been protonated. Therefore, it can be considered theoretically that the sensitivity is not affected by the pH of the mobile phase. However, the experimental results confirm that (1) the sensitivity of mass spectrometry can be significantly enhanced by the acidifying mobile phase; and (2) the *m*/*z* 117 ion was not observed in the MS scan. These phenomena confirmed that the in-source fragmentation of dicofol is accompanied by a protonated transition state resulting in trichloromethyl leaving as a trichloromethane molecular. Nevertheless, why is not hydroxyl protonated and dehydrated?

According to the results of electron density analysis, *p*-conjugation of the two lone oxygen atom pairs with the two aromatic rings reduced the electron density of oxygen atoms to a level insufficient to capture H^+^, preventing protonation of the hydroxyl group. Moreover, as a strong electronegative group, trichloromethyl was unable to effectively capture H^+^ due to steric hindrance and *p*-conjugation. Due to the orienting effect of the chlorine atoms, the parasite of chlorine atoms on two aromatic rings became the best electrophilic attack site of H^+^ and was inferred to be the protonation site of the dicofol molecule.

**Figure 3:**
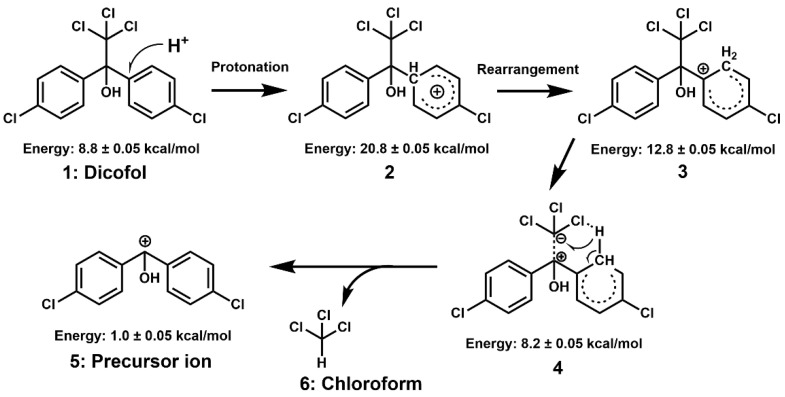
Chemical process of dicofol in-source fragmentation reaction.

The calculation of internal particle energy contributes to inferring the mechanism of in-source fragmentation. As shown in Figure 3, the process is as follows: (i) H^+^ may easily attack the *para* site of the chlorobenzene structure under the little energy provided by the nozzle voltage (Figure 3, Structure 1), which leads the benzene ring to be charged and to produce the transition states with structural changes. However, this step only increased the molecular energy from 8.8 kcal/mol to 20.8 kcal/mol (Figure 3, Structure 2); (ii) The H atoms at the protonation site rearranged to ortho and formed tert-butyl carbocation, which is an intermediate reaction with decrease molecular total energy from 20.8 kcal/mol to 12.8 kcal/mol (Figure 3, Structure 3); (iii) The rearranged H atom formed a stable hexatomic ring with one chlorine atom of trichloromethyl through a hydrogen bond.

Meanwhile, the electron-withdrawing effect of the hydroxyl and the recovery conjugation tendency of the benzene ring induced the positive charge center shift to the benzyl carbon, thereby strengthening the bond energy between the benzyl carbon and the hydroxyl and weakening the bond energy between the benzyl carbon and the trichloromethyl. As a result, the molecular energy is further released from 12.8 kcal/mol to 8.2 kcal/mol (Figure 3, Structure 4); (iv) The hydrogen and weak bonds of the hexatomic ring were broken, which produced chloroform (Figure 3, Structure 6) and the precursor ion (Figure 3, Structure 5). As a result, the internal energy was reduced to 1.0 kcal/mol.

**Figure 4:**
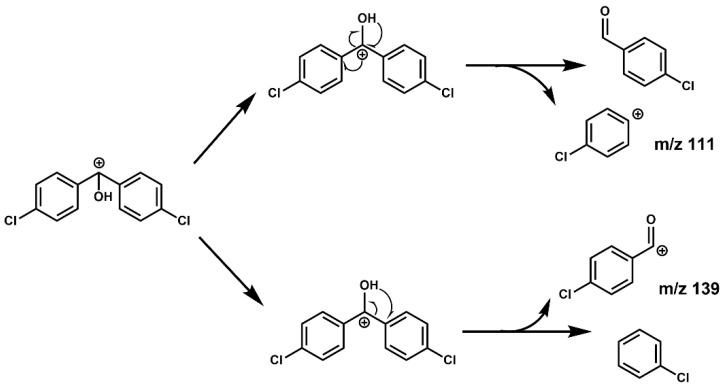
CID process of the precursor ion *m*/*z* 251.1.

The CID of the precursor ion in the collision cell mainly produced two positive ions. As shown in Figure 4, first, the benzyl of the precursor ion was broken by collision. As a result, it produced free radicals dehydrogenated into a positive ion of the 4-chlorobenzaldehyde, absent the H atom on the aldehyde group or a tropylium cation (*m*/*z* 139) by rearrangement. Second, due to the induced effect of chlorobenzene, the positive charge center of the precursor ion rearranged to the *para* site again, which weakened and broke the bond energy between the benzene ring and methyl and finally produced the positive ion of the chlorobenzene absent the *para* site H atom (*m*/*z* 111). In addition, the charge and the hydrogen atom on hydroxyl are rearranged into 4-chlorobenzaldehyde molecules, and then 4-chlorobenzaldehyde may break and rearrange, similar to *m*/*z* 139. Therefore, we concluded that the higher yield of *m*/*z* 139 than *m*/*z* 111 might be the reason for its greater abundance.

### 2.6. Extraction Method of Dicofol Based on HPLC-MS/MS

A method was developed for the analysis of dicofol residues in environmental water. The standard substance of dicofol was added to blank samples at four levels (0.1, 1, 10, and 100 μg/L). Extraction and analysis results are shown in Figure S13 and Table 1. The peak areas of the product ions from MS/MS were used to calculate the recovery, linearity, limit of quantification (LOQ), and limit of detection (LOD). The LOQ of dicofol in environmental water was 0.1 μg/L, and the LOD was 0.05 μg/L. The average recoveries were 68% (RSD = 8.6%) for 0.1 μg/L, 64% (RSD = 5.4%) for 1 μg/L, 77% (RSD = 7%) for 10 μg/L, and 86% (RSD = 11.7%) for 100 μg/L, respectively. The linear regression equation was y = 955x − 325.6 (R^2^ = 0.9999). In addition, the LOQ and LOD of dicofol in pure solvent (acetonitrile) were 0.1 μg/L and 0.02 μg/L, respectively, and the linear regression equation was y = 1051x − 460 (R^2^ = 0.9999). The result, considered acceptable, states an excellent performance of the method.

In previous reference studies, gas chromatography or GC-MS usually performed dicofol analysis. For example, A. Peris used GC-MS to study dicofol residue in sediments of a river and obtained a LOQ of 0.17 μg/L [18,19]. Hui Wang et al. extracted dicofol from aquatic products and analyzed it with GC-ECD with a LOQ of 0.4 μg/kg [31]. The comparison fully demonstrated that the HPLC-MS/MS method established in this study was suitable for analyzing dicofol in environmental substrates and was superior to the previous methods. This study provided a new solution for the rapid and simple analysis of dicofol.

### 2.7. Analysis Results in Environmental Water Samples

This study was used to monitor dicofol in 321 real environmental water samples, including surface and groundwater. The water samples were collected from nine provinces in China in 2021. All of them were agricultural areas where dicofol had been widely applied before being banned. The results showed that no dicofol was detected in any sample in this survey. Although no residual dicofol was found in this study, it does not mean that the method we established has lost its value. On the contrary, this study can promote the development of environmental governance in a better direction. Simultaneously, the significance of this study is not only the analysis of a single compound of dicofol but also to provide solutions for the detection of more compounds that are difficult to analyze through this study.

## 3. Materials and Methods

### 3.1. Regents and Materials

The chemicals and solvents used to prepare standard solvents and mobile phases of instruments must be of HPLC grade. Other reagents used for extraction and cleanup were at least analytical reagent (AR) grade. Chromatography-grade acetonitrile, methanol, and formic acid were purchased from Sigma-Aldrich (Steinheim, Germany). Sodium chloride (NaCl), anhydrous magnesium sulfate (MgSO_4_), ammonium acetate (CH_3_COONH_4_), ammonia (HH_3_·H_2_O), and analytical grade acetonitrile were purchased from Sinopharm Chemical Reagent Co., Ltd. (Shanghai, China). Primary, secondary amine (PSA, 40 μm), and 0.22 μm nylon syringe filters were purchased from Agela Technologies Inc. (Agela, Tianjin, China). Dicofol (99.7% purity) reference materials were obtained from Alta Scientific Co., Ltd. (Tianjin, China). Ultra-pure water was obtained using a Milli-Q reagent water system (Bedford, MA, USA).

### 3.2. Instrument and Parameters Test

The dicofol reference material was dissolved in HPLC-grade methanol at concentrations of 250, 500, and 1000 μg/L, respectively. A high-performance liquid chromatography (HPLC) system (Agilent 1290 InfinityII, Santa Clara, CA, USA) tandem triple quadrupole mass spectrometer (Agilent 6470) equipped with an electrospray ionization (ESI) source was used in the study. A reversed-phase column (Poroshell 120 EC-C_18_, 2.1 × 50 mm, 1.9 μm, Agilent Technologies Inc., Santa Clara, CA, USA) was used to separate the mixture. The column temperature was selected at 30 °C. The injection volume was 5 μL. The detection was operated in multiple reaction monitoring (MRM) to identify the specific precursor ion and product ions inside retention time windows. A series of software, including Data Acquisition, Qualitative Navigator, and QQQ Quantitative Analysis, were used for instrument control and data acquisition.

Employing fractional factorial experiment design, seven crucial parameters, including drying gas temperature and flow rate, nebulizer pressure, sheath gas temperature and flow rate, capillary voltage, and nozzle voltage, were optimized using the design experiment approach. As the organic component of the mobile phase, acetonitrile and methanol were used, while the formic acid, ammonium acetate, and ammonia dissolved in water comprised the inorganic component. Extending the general setting to empirically higher and lower limits was used to test a series of levels for each factor. As the sensitivity of the instrument was dependent on signal response and background noise, peak area, and signal-to-noise ratio (S/N) were used as the reference data. In each trial, injections were made in triplicate.

### 3.3. Theoretical Calculation

In support of the density functional theory (DFT) calculations, dissociation reactions resulting from the protonation of trichloromethyl due to in-source fragments in ESI were studied. The Fukui function is used as the primary basis for calculations, and the Gaussian 16 suite of programs was used to perform quantum chemical calculations and generate color-filled maps of three-dimensional electron density. The optimum geometry of dicofol in the mobile phase at the elution time and the stationary points on the potential surface were obtained at the B3LYP/6-31+G(d) level of theory. Based on the optimum geometry, natural bond orbital (NBO) charges analyses at the same level were then used [32]. The Fukui value of electron distribution was obtained by the Multiwfn [33]. The position of electrophilic acts as the protonation site of the dicofol molecule takes place as follows:

$$f_{k}^{-}=q_{k}\left(N\right)-q_{k}\left(N-1\right)$$

$$f_{k}^{+}=q_{k}\left(N+1\right)-q_{k}\left(N\right)$$

where $f_{k}^{-}$ is the electrophilic attack, $f_{k}^{+}$ is the nucleophilic attack, *q_k_* is the electronic population of atom *k* in a molecule, and *N* is the total number of electrons in the system. It is more convenient to represent the Fukui values around each atomic site into a single value that characterizes the electronic population in a molecule. Dual descriptor Δ*f*(*r*) [28,34] for the calculation of nucleophilicity and electrophilicity is defined as the difference between the nucleophilic and electrophilic Fukui function and is given by the equation:

$$\Delta f(r)=f_{k}^{+}-f_{k}^{-}$$

### 3.4. Extraction Test and Method Velidation

A recovery test was performed to establish an extraction method for dicofol based on HPLC-MS/MS. Environmental water was selected as the matrix for the tests as it is the most likely dicofol carrier in the environment. The water was collected in a mountain stream to ensure free of dicofol.

A 10.00 ± 0.05 mL sample was weighed in a 50 mL Teflon centrifuge tube. Dicofol standard substance was added to blank samples at four levels (0.1, 1, 10, and 100 μg/kg or μg/L). After shaking and quiescence for 30 min, 10 mL of n-hexane (AR) was added as extracting solvent, and an automatic shaker mixed the tube for 10 min at 2000 rpm. Then the sample was centrifuged (4000 rpm, 5 min). Next, 1.5 mL of supernatant was transferred from each tube into a 2 mL polypropylene centrifuge tube with preloaded cleaner. According to the pre-experiment results and the characteristics of the matrix, the cleaner was selected as 50 mg PSA and 150 mg anhydrous MgSO_4_. All 2 mL tubes were shaken vigorously by vortex for 1 min and then centrifuged at 5000 rpm for 5 min. Finally, supernatants were filtered through 0.22 μm nylon syringe filters and transferred to an autosampler vial for HPLC-MS/MS.

In terms of recovery, repeatability, LOQ, LOD, linearity, and matrix effects, the analytical performances of dicofol in environmental water were assessed according to recovery test results. The LOQ and LOD were established as the lowest validated concentration that can be measured with acceptable accuracy and precision, in accordance with the European Commission’s recommendation SANTE/12682/2019 [35]. The detailed definition and calculation methods of other parameters have been reported in a previously published by [2].

### 3.5. Analysis of Environmental Water Samples

A total of 321 environmental water samples, including surface and groundwater, were collected from agricultural areas in nine provinces in China in 2021. Dicofol was analyzed using the method described in Section 3.4. The external standard method was used as a quantitative method.

## 4. Conclusions

Dicofol has long been difficult to be analyzed by HPLC-MS/MS because it cannot be ionized by ESI. In response to this technical bottleneck, in this study, the HPLC-MS/MS method for dicofol was developed and applied for the first time to analyze environmental samples based on in-source fragmentation. Furthermore, a comprehensive analysis of the chemical behavior of dicofol in the ESI source was conducted.

Two prerequisite conditions for organic molecules’ in-source fragmentation were proposed to analyze the mechanism. One is that ions need sufficient internal energy to fragment [36], and the other is that the covalent bond can easily cleavage and produce a stable structure. Internal energy release from high to low levels is the driving force of in-source fragmentation, as the molecular structure tends to be stable. In addition, it is believed that pH catalysis plays a crucial role in the regulation of in-source fragmentation.

In-source fragmentation has been a significant negative factor contributing to the low detection ability of target compounds in HPLC-MS/MS for a very long time. However, through this study, we believe that in-source fragmentation can provide new light for high-sensitivity analysis of environmental pollutants that were previously thought to be difficult to detect using HPLC-MS/MS. We anticipate that this study will establish HPLC-MS/MS methods for additional environmental pollutants, as well as analyze and control the mass spectrometry behavior. In the meantime, we anticipate that this study will inspire additional research fields, including identifying and analyzing degradation or metabolites, analytical chemistry, omics, and mass-spectrometry technique.

## Figures and Tables

**Table 1:** Recoveries, RSDs for dicofol in environmental water at four spiked levels.

Level (μg/L)	Recovery (%)						RSD (%)
	1	2	3	4	5	Average	
0.1	79	73	67	68	56	68	8.6
1.0	61	59	62	67	72	64	5.4
10.0	77	78	65	85	78	77	7.0
100.0	86	75	73	97	98	86	11.7

## Data Availability

All data are available upon reasonable request from the corresponding author.

## Supplementary Materials

The following supporting information can be downloaded at: https://www.mdpi.com/article/10.3390/molecules28093765/s1. The introduction of dicofol and all original chromatogram or mass spectrogram can be found in Supplementary Materials.
