# Supplementary material for: Spontaneous In-Source Fragmentation Reaction Mechanism and Highly Sensitive Analysis of Dicofol by Electrospray Ionization Mass Spectrometry
Source: Molecules. 2023 Apr 27;28(9):3765. doi: 10.3390/molecules28093765 (PMC10180504; doi:10.3390/molecules28093765)
Supplement: Supplementary file 1 [file molecules-28-03765-s001.zip › molecules-2348271-supplementary.pdf]

Supplementary Material

Spontaneous In-Source Fragmentation Reaction Mechanism and Highly Sensitive Analysis of Dicofol by Electrospray Ionization Mass Spectrometry

Jun Xie [1,2], Yage Guo [1], Yongqiang Ma [2], Hongyun Jiang [1], Lan Zhang [1], Liangang Mao [1], Lizhen Zhu [1], Yongquan Zheng [3] and Xingang Liu [1,*]

[1] State Key Laboratory for Biology of Plant Diseases and Insect Pests, Institute of Plant Protection, Chinese Academy of Agricultural Sciences, Beijing 100193, China; xxjj2410@foxmail.com (J.X.)

[2] Department of Applied Chemistry, College of Science, China Agricultural University, Beijing 100193, China

[3] Shandong Engineering Research Center for Environment-Friendly Agricultural Pest Management, College of Plant Health and Medicine, Qingdao Agricultural University, Qingdao 266109, China

* Correspondence: liuxingang@caas.cn; Tel./Fax: +86-010-62815938

S1 Introduction of Dicofol

Trade Name: Carbax, Mitigan, Acarin, Mitex, Kelthane, Hilfol

CAS Number: 115-32-2

CAS Name: 4-chloro-α-(4-chlorophenyl)-α-(trichloromethyl)benzenemethanol

IUPAC Name: 2,2,2-trichloro-1,1-bis(4-chlorophenyl)ethanol

Molecular Formula: $C_{14}H_9Cl_5O$

Molecular Mass: 370.49

Melting Point: 370.5 °C

Boiling Point: 190.3 °C

Solubility: 0.8 mg/L in water, 400 g/L in Acetone, more than 1000 g/L in alcohols

Octanol-water partition coefficient (Kow): 2.0×10^4 *at pH 7, 20 °C*

Log Kow: 4.3

Physical State: Pure dicofol is a white crystalline solid. Technical dicofol is a red-brown or amber viscous liquid with an odor like fresh-cut hay.

Toxicity:

The acute oral LD50 in rats is 575 to 960 mg/kg; in rabbits and guinea pigs it is 1810 mg/kg; and in mice it is 420 to 675 mg/kg. The dermal LD50 in rats is 1,000 to 5,000 mg/kg, and in rabbits it is between 2,000 and 5,000 mg/kg. The inhalation LC50 (4-hour) in rats is greater than 5 mg/L. Exposure of animal species (e.g., mice, rats, dogs) to dicofol caused liver enlargement/damage, damage to the kidney and adrenals, and eventually death.

Dicofol is absorbed through ingestion, inhalation, or skin contact. Symptoms of exposure include nausea, dizziness, weakness, vomiting from ingestion or respiratory exposure, skin irritation or rash from dermal exposure, and conjunctivitis from eye contact. Poisoning may affect the liver, kidneys, or CNS. Overexposure by any route may cause nervousness and hyperactivity, headache, nausea, vomiting, unusual sensations, and fatigue. Very severe cases may result in convulsions, coma, or death from respiratory failure. Dicofol is a moderate skin and eye irritant. Since dicofol is stored in fatty tissues, intense activity or starvation may mobilize the pesticide, resulting in the reappearance of toxic symptoms long after actual exposure.

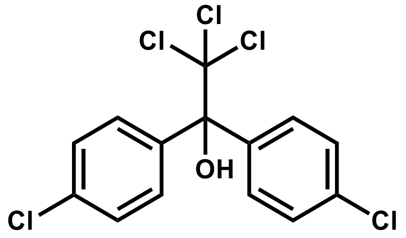

Figure S1. The molecular formula for dicofol

S2 The original chromatogram or mass spectrogram

S2.1 Confirmation of In-source Fragmentation in ESI source

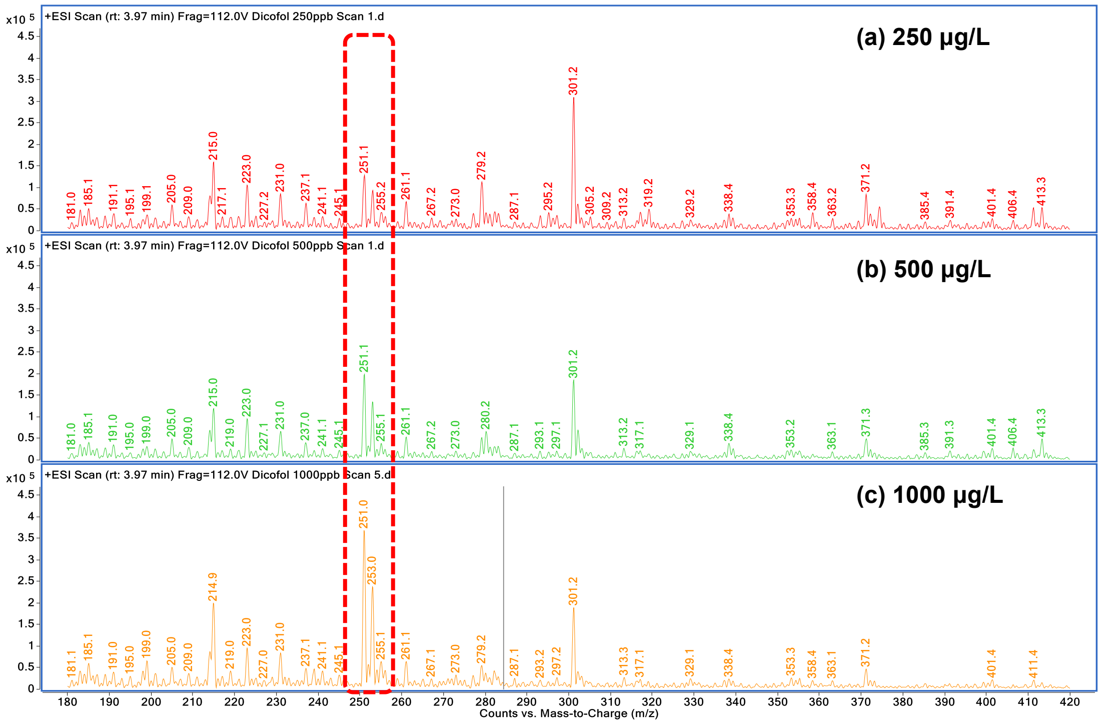

Figure S2. Mass scan spectrogram of the dicofol precursor ion for different concentrations

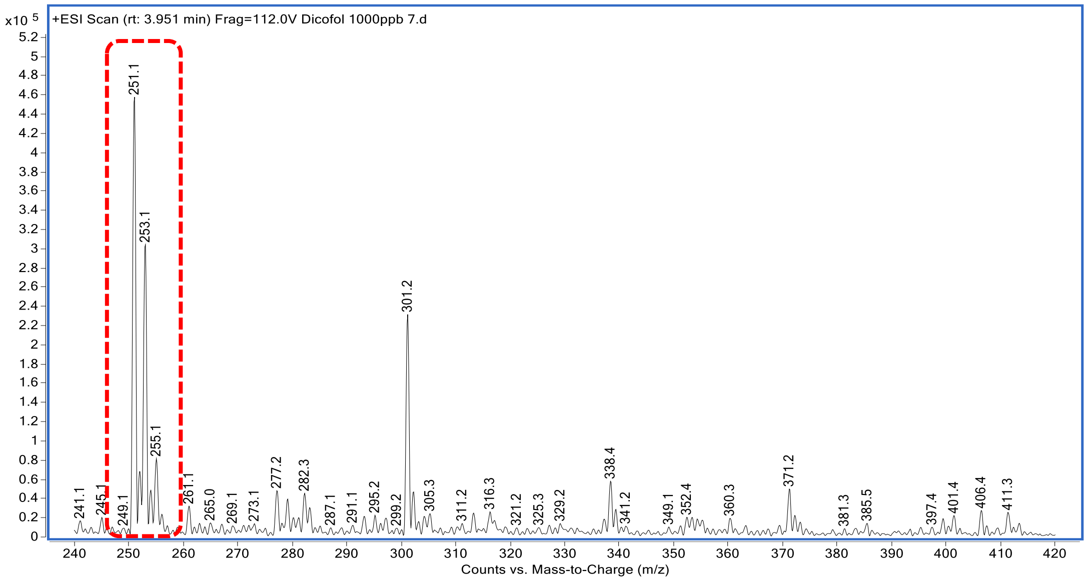

Figure S3. Mass scan spectrogram of the dicofol precursor ion for 1000 μg/L

The response of the mass spectrogram signal *m/z* 251 was positively correlate with the concentration of the standard solutions. *m/z* 251, *m/z* 253 and *m/z* 255 are three continuous mass spectral signals with mass charge ratio difference of 2.

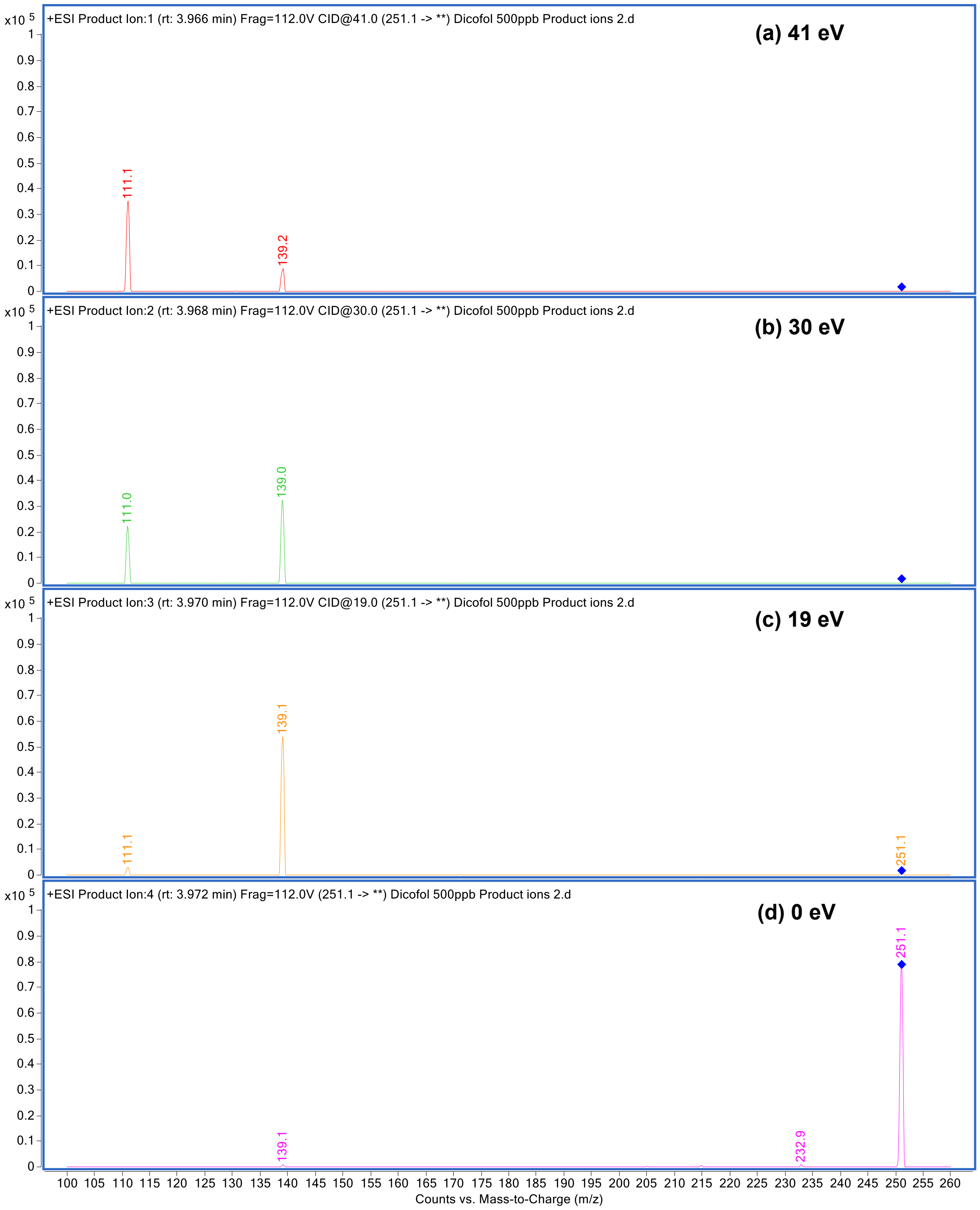

Figure S4. Product ions for different collision energies for CID

In addition to the weak dehydration product ion *m/z 232.9*, two stronger product ions *m/z 139.1* and *m/z 111.1* are readily produced in CID.

S2.2 Influences of critical source parameters

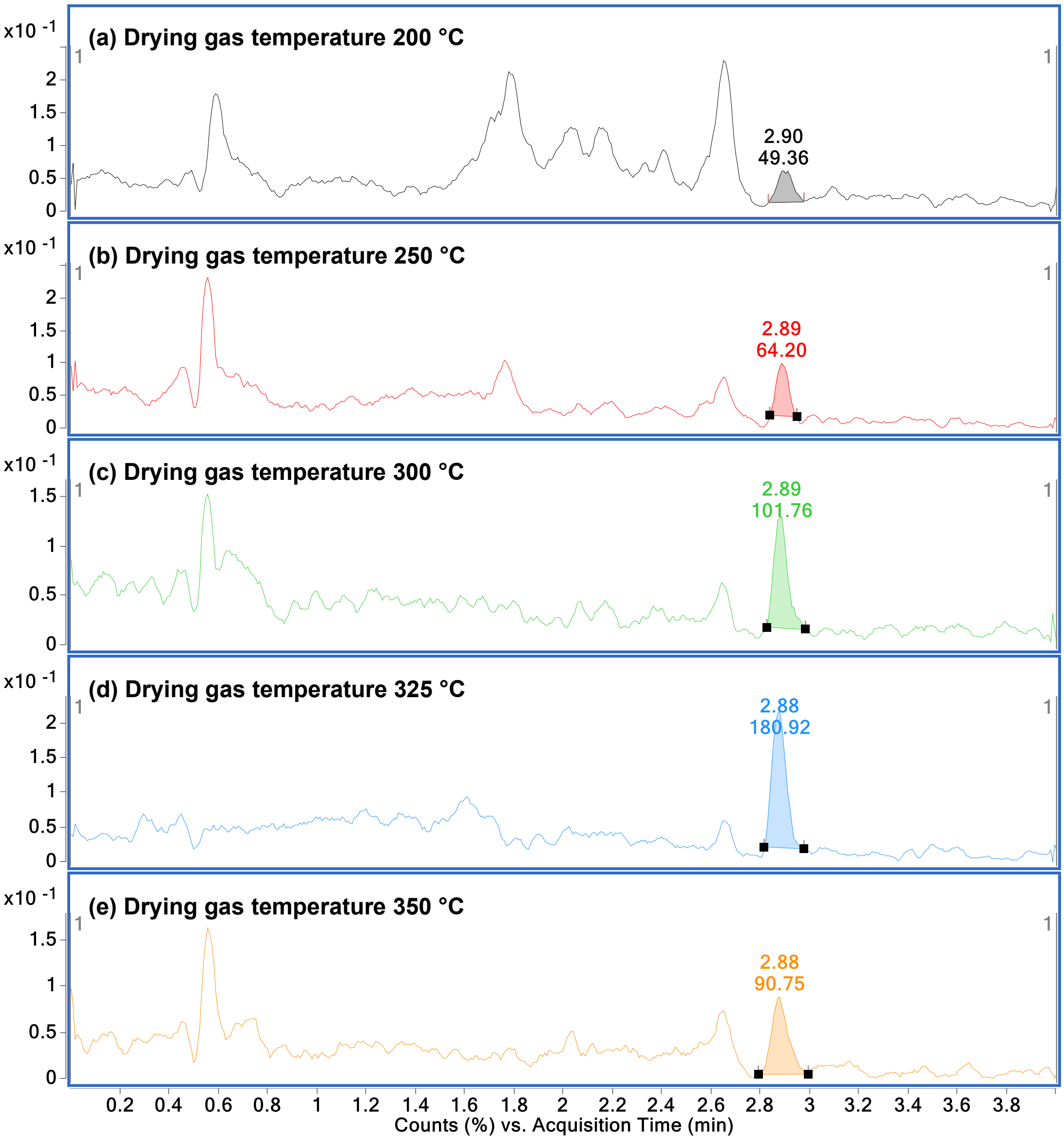

Figure S5. Impact of drying gas on detection capability

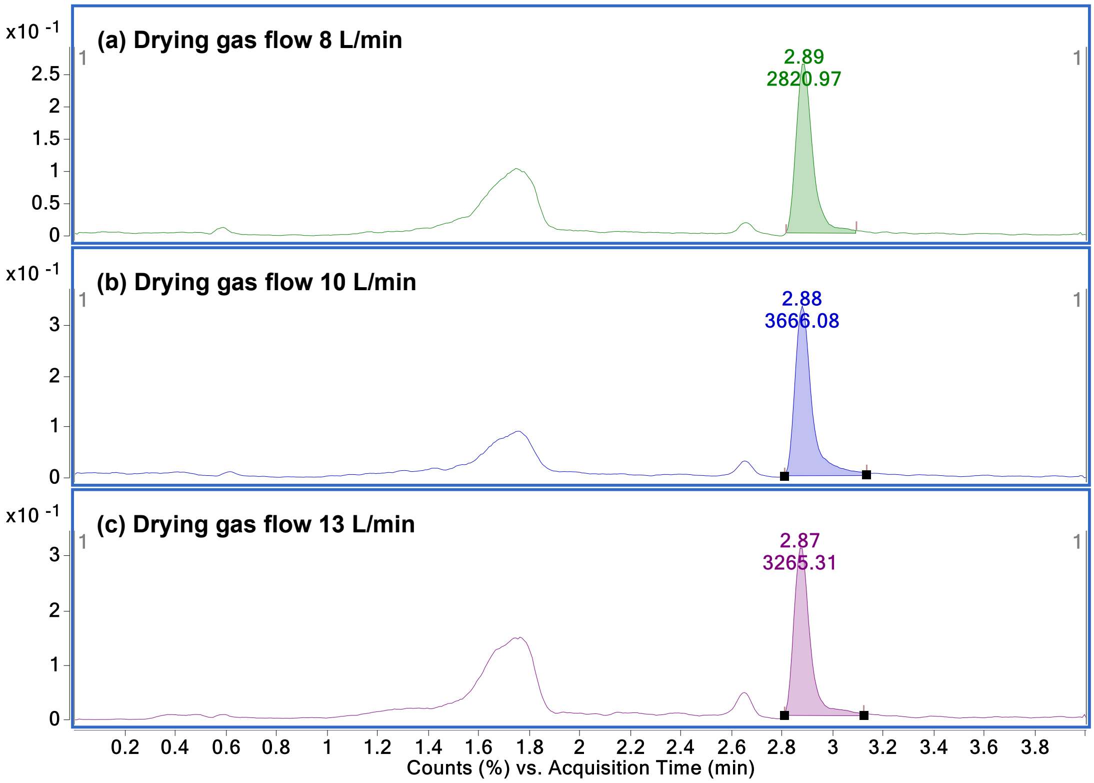

Figure S6. Impact of drying gas flow on detection capability

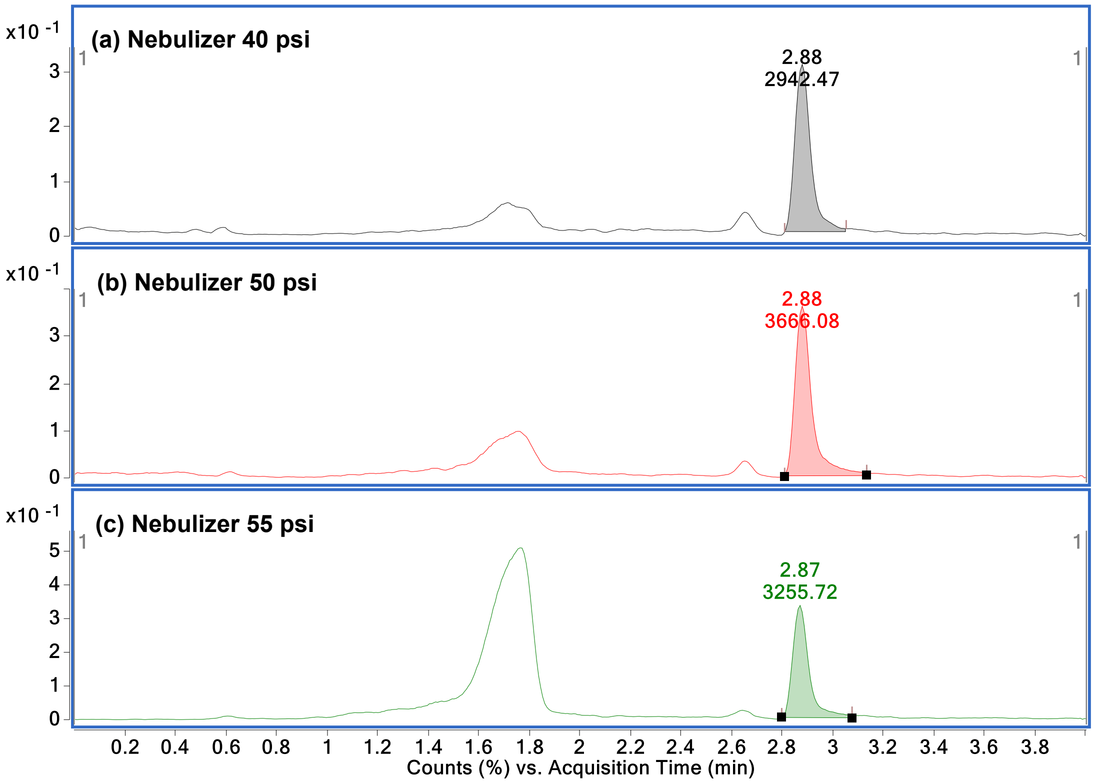

Figure S7. Impact of nebulization pressure on detection capability

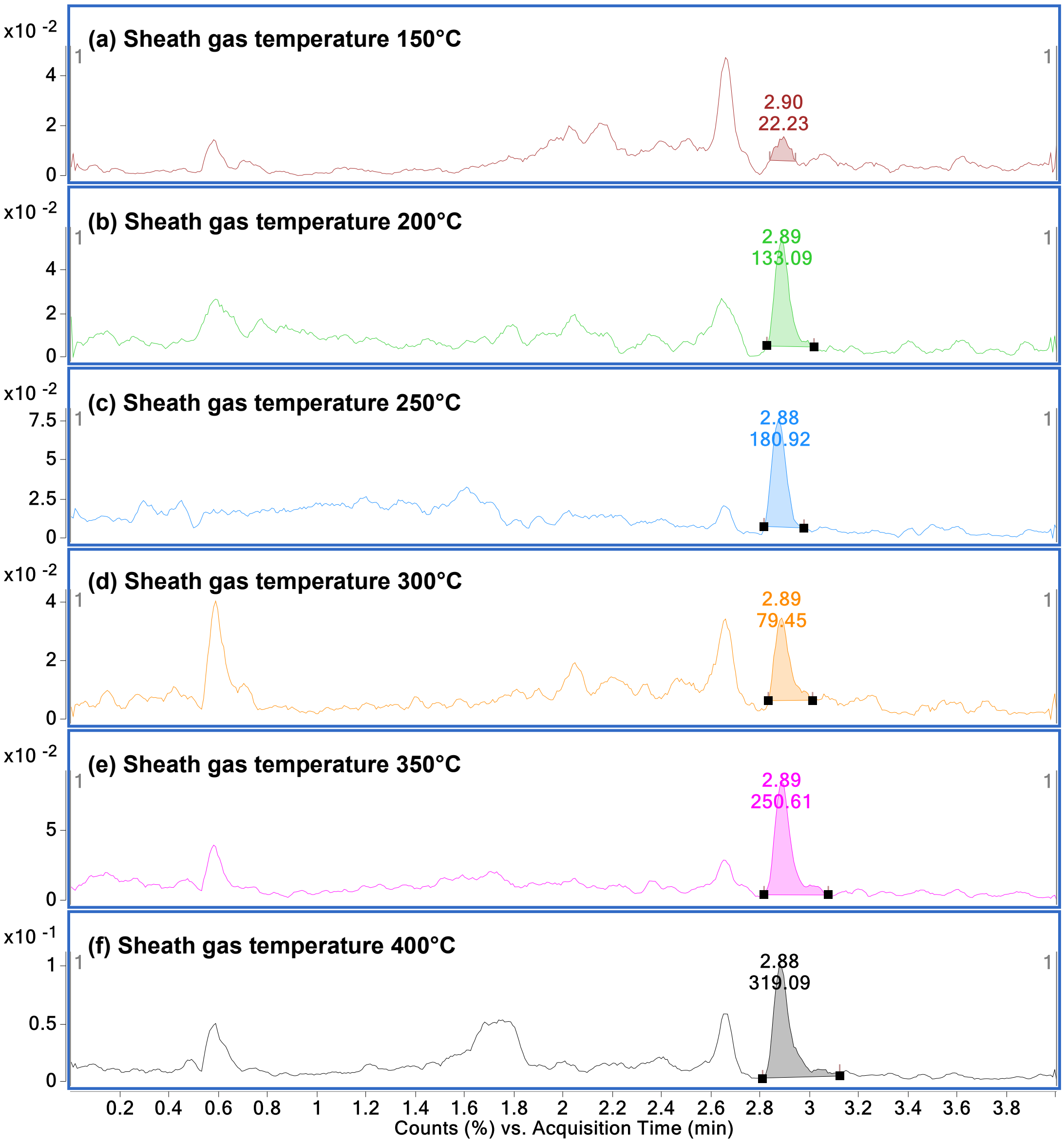

Figure S8. Impact of sheath gas temperature on detection capability

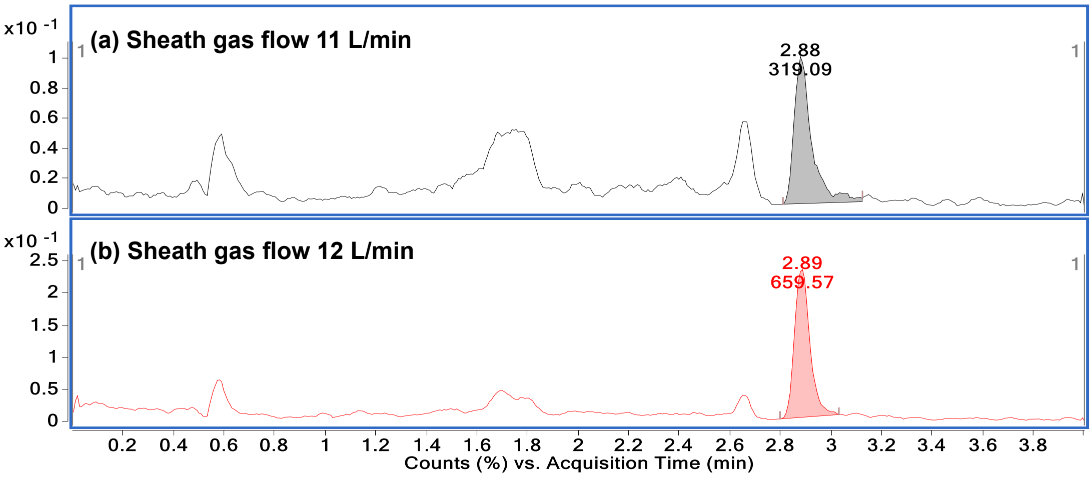

Figure S9. Impact of sheath gas flow on detection capability

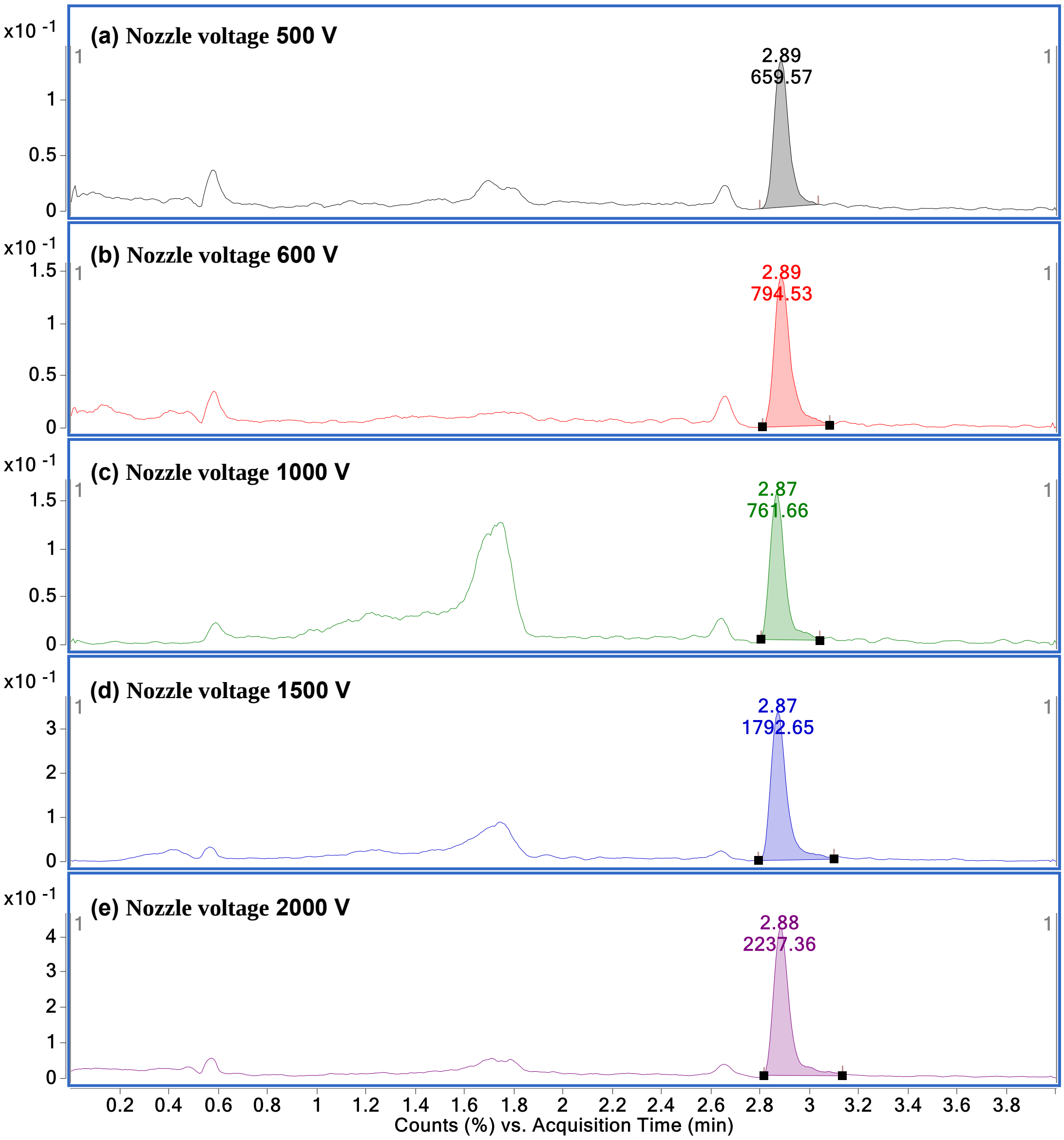

Figure S10. Impact of nozzle voltage on detection capability

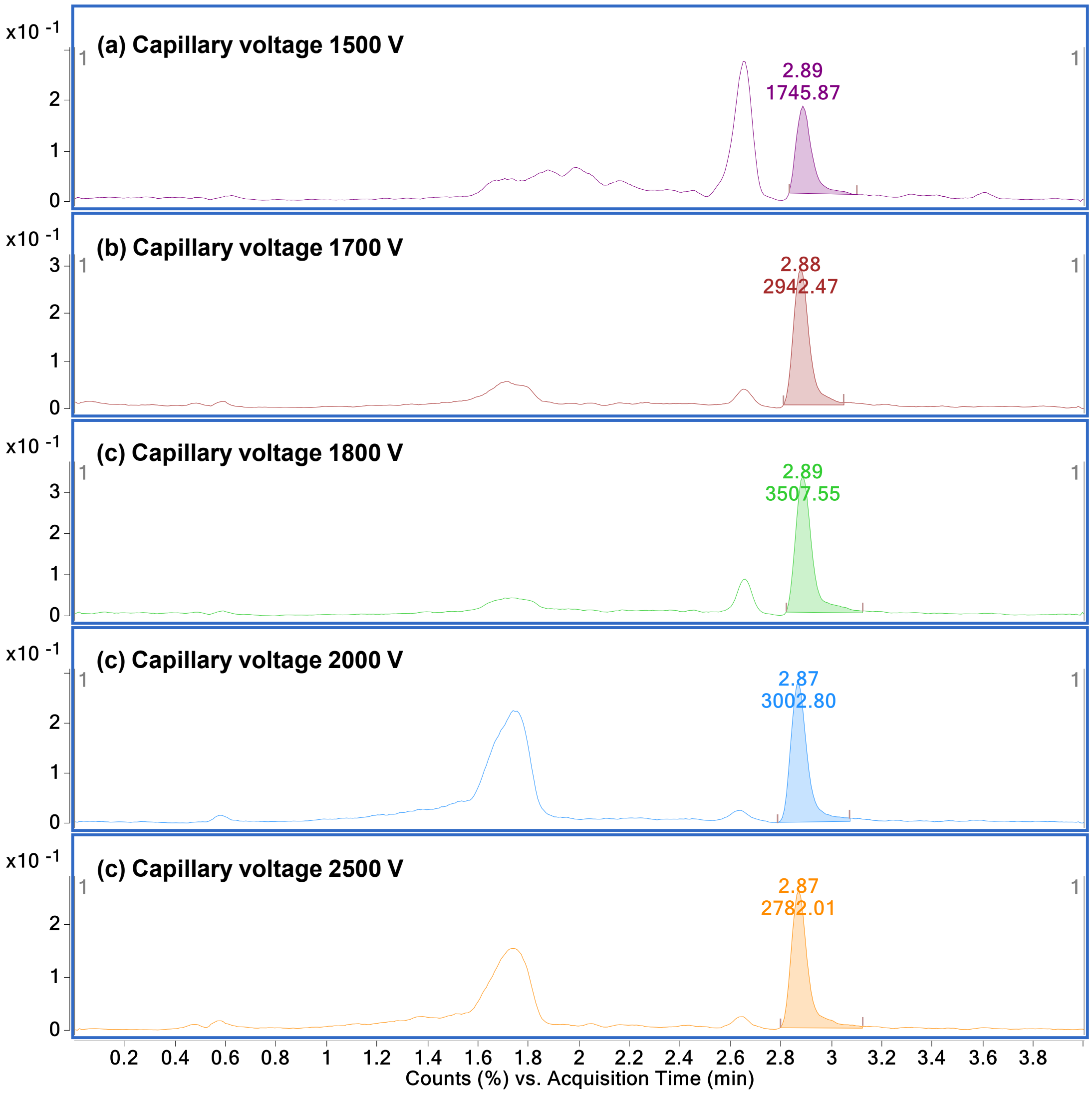

Figure S11. Impact of capillary voltage on detection capability

S2.3 Influence of chromatographic conditions

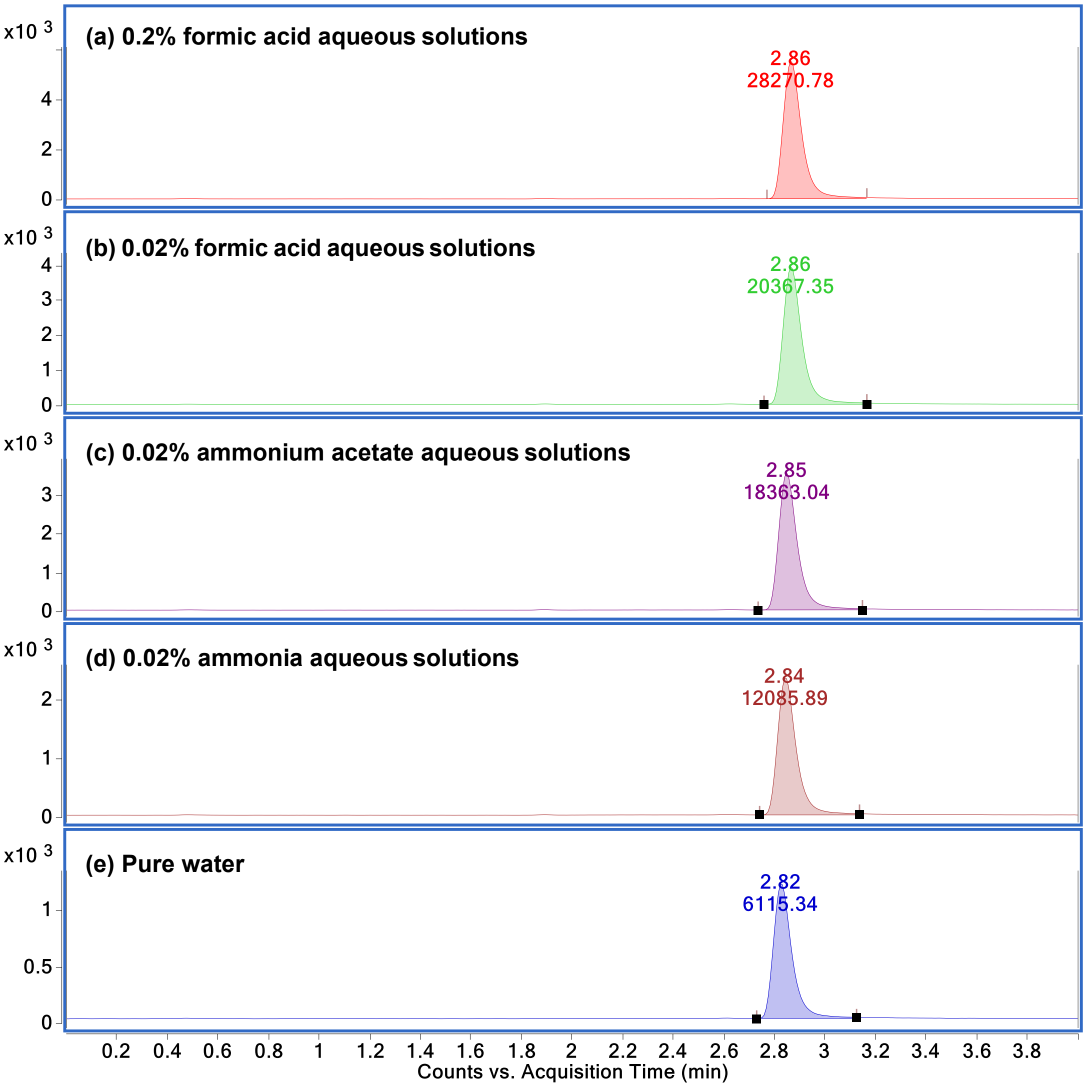

Figure S12. Impact of mobile phase (water phase) additive on detection capability

S2.4 Extraction method of dicofol based on HPLC-MS/MS

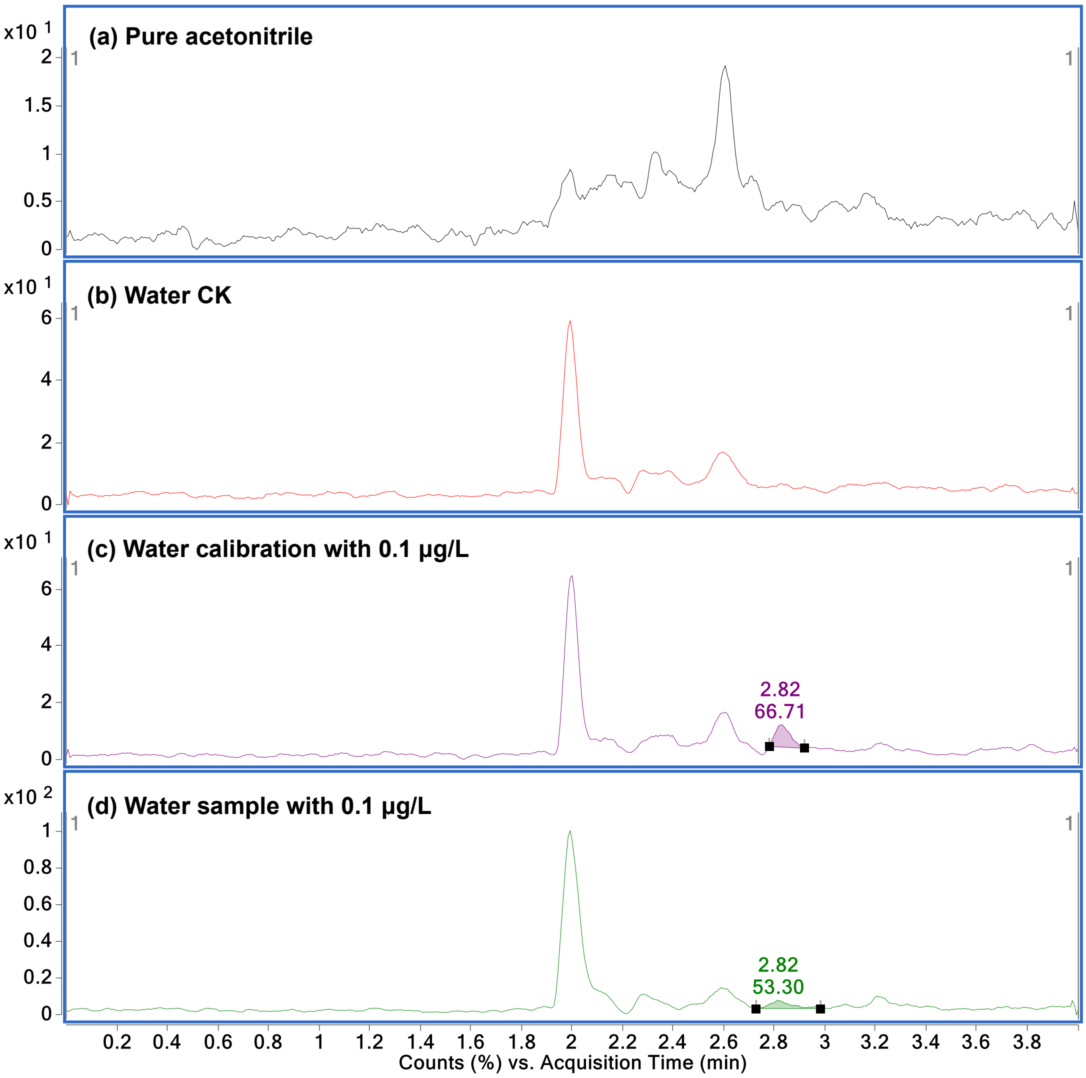

Figure S13. Typical chromatograms of dicofol
